# The effects of a treatment combination of anti-VEGF injections, laser coagulation and cryotherapy on patients with type 3 Coat’s disease

**DOI:** 10.1186/s12886-017-0469-4

**Published:** 2017-05-22

**Authors:** Songfeng Li, Guangda Deng, Jinghua Liu, Yan Ma, Hai Lu

**Affiliations:** Beijing Ophthalmology and Visual Science Key Laboratory, Beijing Tongren Eye Center, Beijing Tongren Hospital,Capital Medical University, No. 1 Dongjiao Min Xiang, 100730 Beijing, People’s Republic of China

**Keywords:** Coats’disease, Treatment, Retinal detachment, Ranibizumab

## Abstract

**Background:**

To examined the curative effect of vitreous injection with ranibizumab,laser coagulation and cryotherapy in treating stage 3 Coats’ disease with exudative retinal detachment.

**Methods:**

Seventeen patients with stage 3 Coats’ disease were enrolled in the study. All eyes were treated with vitreous injection of ranibizumab as initial treatment, and subsequent treatment depended on the absorption of subretinal fluid, Including cryotherapy and laser photocoagulation. Repeat treatment for the two treatment intervals occurred in ≥1 month. The mean follow-up time was 24.12 ± 5.99 months. The main data evaluation and outcome measurements included the patient’s vision, intraocular pressure(IOP), optical coherence tomography (OCT), slit lamp examination, indirect ophthalmoscopy, color Doppler imaging (CDI) and color fundus image analysis. The following variables were compared between groups: abnormal vascular changes, subretinal fluid and exudate absorption, retinal reattachment and complications. The final follow-up results were used to determine the effectiveness of treatment.

**Results:**

Of the 17 patients included, 88.24% were male and 11.76% were female. Visual acuity was less than 0.02 in 12 eyes before surgery and 8 eyes after surgery. Visual acuity improved in 7 eyes, accounting for 41.18% of cases, and remained unchanged in 7 eyes, accounting for 41.18% of cases. Three patients were too young to undergo the operation, accounting for 17.65% of cases. The best vision was 0.1. Patients were treated 1 to 5 times for an average of 2.82 ± 0.95 times each. There was no statistically significant difference (*t* = 1.580, *p* = 0.135) between the preoperative and postoperative intraocular pressures. However, there was a statistically significant difference between the preoperative and postoperative retinal detachment height (2- related samples Wilcoxon signed rank test with z = 3.517, *p* = 0.000). The results further showed that all patients had different degrees of subretinal fluid absorption, and some of the new blood vessels subsided. All patients were successfully treated with laser and cryosurgery. No ocular or systemic complications were observed during follow-up.

**Conclusions:**

Intravitreal ranibizumab (IVR), laser coagulation and cryotherapy were effective in the treatment of Coats’ disease with exudative retinal detachment.

**Trial registration number:**

We retrospectively registered our study, The trial registration number (TRN) is ChiCTR-ONC-17011161 and date of registration is April 16, 2017.

## Background

In 1908, Coats first described a case of male monocular retinal hemorrhage in a child with retinal telangiectasia [1]. Shields et al. defined this disease as “idiopathic retinal telangiectasia with retinal detachment or exudative retinal detachment [2].” Coats’ disease typically manifests as idiopathic retinal vascular abnormalities, retinal telangiectasia, and retinal, interretinal, and subretinal infiltration, which can lead to severe exudative retinal detachment.

Coats’ disease commonly occurs in young children. Therefore, it is very difficult to detect in the early stage, and patients often present with higher than stage 3 when diagnosed. For these patients, treatment with retinal laser photocoagulation and cryotherapy alone is not ideal. In recent years, intravitreal injection of anti-vascular endothelial growth factor drugs has been widely used in age-related macular degeneration, diabetic retinopathy, macular edema, retinal central vein obstruction macular edema and other diseases. Studies have also shown that Coats’ patients have high expression levels of intravascular vascular endothelial growth factor (VEGF) [3–6]. These findings suggest that intravitreal injection of anti-VEGF drugs may help to reduce the VEGF concentration, promote subretinal fluid and exudate absorption and stabilize against abnormal proliferation or degradation of vasculature. Additionally, a few studies have investigated anti-VEGF drug treatment in Coats’ disease and achieved good efficacy in the cases reported [3–6], although the overall sample size has been small. We retrospectively analyzed the use of anti-VEGF drugs combined with other methods to treat Coats’ disease with the aim of exploring the use, safety, and effectiveness of anti-VEGF drugs in the treatment of Coats’ disease. The results are reported below.

## Objectives and methods

This was a retrospective, non-controlled clinical study. Seventeen patients (17 eyes) with Coats’ disease diagnosed at the Eye Center of Tongren Hospital were included in the study, from April 2013 to september 2016. The participants were aged 2 to 11 years with a mean age of 4.94 ± 2.92 years. All had monocular disease. The study was conducted in accordance with the ethical standards stated in the 1964 Declaration of Helsinki and approved by the Ethics Committee of Beijing Tongren Hospital. Both patients and their parents gave written informed consents after explanation of the nature and possible consequences of the treatment.

All patients underwent color Doppler imaging (CDI), intraocular pressure (IOP) evaluation, indirect ophthalmoscopy, slit lamp microscopy, fluorescein fundus angiography (FFA) and optical coherence tomography (OCT). The children also underwent Retcam fundus photography under general anesthesia. According to the children’s cognitive ability, we sought to improve the eye examination.

The inclusion criteria consisted of initial treatment with no prior history of treatment and fundus and FFA examination consistent with Coats’ disease. The exclusion criteria were retinoblastoma, familial exudative vitreoretinopathy, retinopathy of prematurity, persistent fetal vasculature, intraocular inflammation, or any other disease associated with retinal exudation.

Coats’ disease was staged according to the Shields method [7] as follows: Stage 1: retinal telangiectasia; Stage 2: can be combined with retinal exudation; Stage 3: exudative retinal detachment (stage 3A1 extrafoveal retinal detachment only, 3A2 foveal detachment); stage 3B, total retinal detachment; Stage 4: complete retinal detachment with glaucoma; and Stage 5: end stage. Of the 17 patients, 10 were classified in stage 3a and 7 in stage 3b.

Of the 17 patients, the visual acuity was lightless in 1 case, hand movement (HM) in 3 cases, counting fingers (CF) in 8 cases, and the values ranged from 0.01 to 0.1 in 2 cases. Three patients were too young to complete the examination. All patients had normal IOP before treatment. Fundus examination revealed abnormal expansion of the retinal blood vessels as well as retinal, interretinal and subretinal exudation that may be associated with retinal detachment. FFA examination revealed that typical retinal capillaries and small blood vessels had abnormal expansion.

Ranibizumab was given at a concentration of 10 mg/ml with a single dose of 0.05 ml (corresponding to 0.5 mg ranibizumab). Before treatment, the patients and their families were fully educated about the disease, informed of intravitreal ranibizumab (IVR) treatment for Coats’ disease-related issues and precautions, and asked to sign an informed consent form. IVR alone, IVR combined with retinal photocoagulation, or IVR combined with cryotherapy was applied according to the extent of retinal detachment, abnormal vascular activity around the retina, and intraocular hyperplasia.

All patients were followed up at 1 day, 1 week and 1 month after intravitreal injection. The follow-up time was determined according to the patient ‘s condition, but the follow-up interval was not more than 1 month. During the follow-up period, the patients with subretinal fluid were treated with IVR alone or in combination with cryotherapy. The patients with partial absorption of the subretinal fluid were treated with IVR combined with retinal photocoagulation. Patients showing a majority of absorption of the subretinal fluid were treated with simple retinal photocoagulation. Repeat treatment occurred in two treatment intervals that occurred ≥1 month later.

The main data evaluation and outcome measurements included the following: the patient’s vision, IOP, slit lamp examination, indirect ophthalmoscopy, color fundus image analysis, abnormal vascular changes, subretinal fluid absorption, retinal reattachment and complications. Before treatment, if the visual acuity was ≥0.1, the visual acuity was considered to have improved or decreased if the value was ≥2 lines. Visual acuity was noted as unchanged for vision within 1 line. Before treatment, if the visual acuity was <0.1, visual acuity was considered improved or decreased if the values was ≥0.02. Visual acuity was noted as unchanged if the value was within 0.02 or less. The changes in the macular retinal detachment height were observed according to OCT before and after treatment. For those children who were not eligible for OCT examination and those with higher macular retinal detachment, OCT examination was replaced by the measurement of the retinal detachment height at 3 mm temporal of the optic disc using ophthalmic CDI.

## Results

All patients were followed up for 15 to 35 months. The mean follow-up time was 24.12 ± 5.99 months. The results of this study are shown in Tables 1 and 2. Of the 17 patients, 88.24% were male and 11.76% were female. The visual acuity was less than 0.02 for 12 eyes before surgery and less than 0.02 for 8 eyes after surgery. Visual acuity improved in 7 eyes, accounting for 41.18% of the population, while it was unchanged in 7 eyes, accounting for 41.18%. Three patients were too young to complete the examination, accounting for 17.65% of cases. The best observed vision in this study was 0.1 (Tables 1 and 2).

**Table 1:** Clinical characteristics of the patients

Patient (NO)	Gender	Age (years)	Follow-up Time (months)	Numbers of IVR	Stage	Laser cases	Cryotherapy cases
1	Male	4	**24**	3	3A	1	0
2	Male	11	**20**	2	3A	1	0
3	Male	3	**35**	2	3A	1	2
4	Male	8	**35**	3	3B	1	1
5	Male	5	**22**	2	3B	2	0
6	Male	3	**22**	4	3A	1	0
7	Male	3	**21**	2	3B	1	0
8	Female	3	**32**	4	3B	1	1
9	Male	10	**29**	2	3A	1	0
10	Male	2	**20**	3	3B	1	0
11	Male	4	**24**	5	3A	3	0
12	Male	5	**19**	3	3B	1	0
13	Female	2	**24**	3	3A	5	0
14	Male	5	**30**	3	3B	1	0
15	Male	7	**15**	3	3A	1	0
16	Male	4	20	1	3A	3	0
17	Male	9	18	3	3A	3	0

**Table 2:** Preoperative and postoperative clinical characteristics

Patient (NO)	Vision		IOP (mmHg)		OCTand CDI (μm)	
	Preoperation	Postoperation	Preoperation	Postoperation	Preoperation	Postoperation
1	HM	0.1	14	13	2100	600
2	0.03	0.1	13	12	447	447
3	Not checked	Not checked	19	17	2200	276
4	Lightless	LP	8	8	2380	265
5	CF	CF	9	9	2100	1200
6	CF	0.05	12	13	1700	1000
7	CF	CF	12	12	7900	2300
8	HM	CF	14	9	3000	468
9	HM	0.05	13	12	1349	1114
10	Not checked	Not checked	9	9	7900	2000
11	CF	CF	8	9	958	781
12	CF	CF	10	11	2900	2200
13	Not checked	Not checked	9	10	1600	560
14	CF	0.05	15	11	768	236
15	0.07	0.1	13	12	919	779
16	CF	CF	9	9	1005	910
17	CF	0.02	12	12	960	116

*HM* hand movement, *LP* light perception, *CF* counting fingers, *IOP* intraocular pressure

**Fig. 1:**
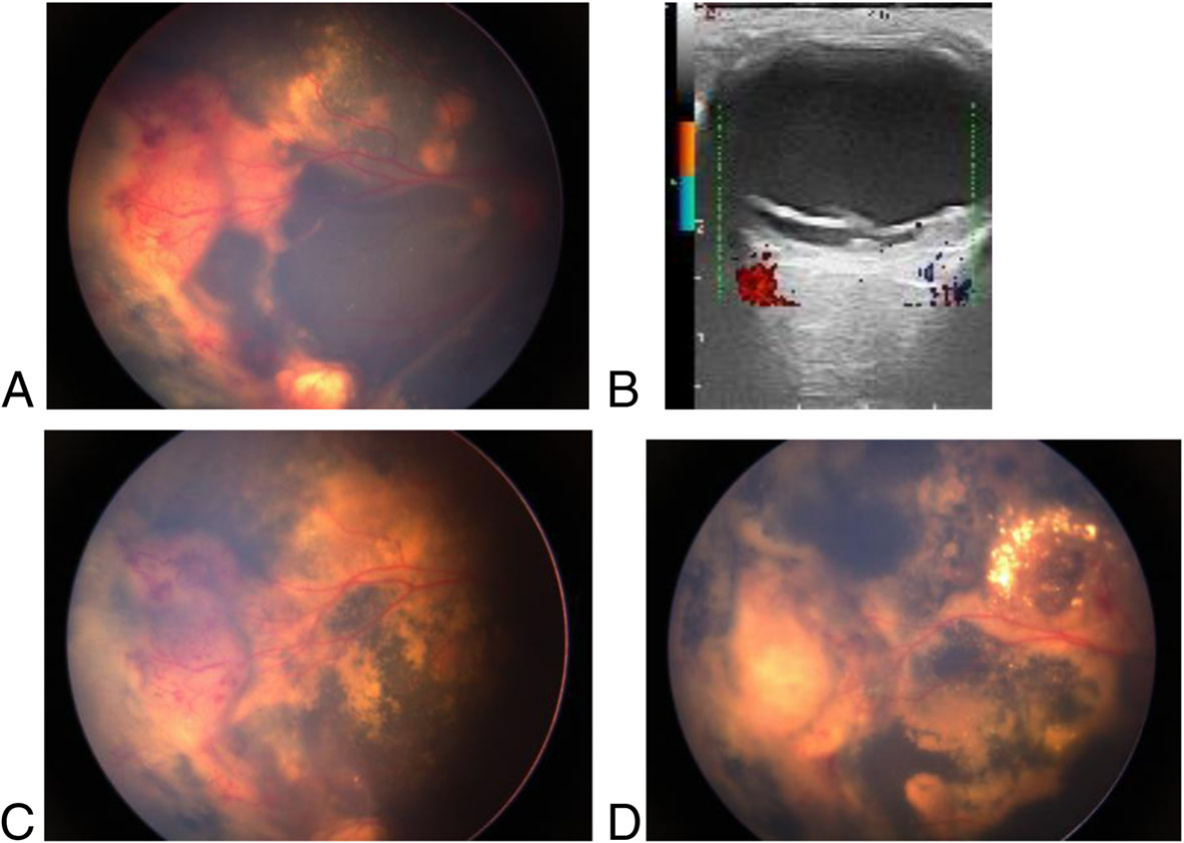
A 8-year-old boy with Coats’disease of right eye. The Retcam imaging of fudus (**a**) and CDI (**b**) shows at baseline shows total retinal detachment before IVR.In the temporal peripheral retina, the presence of abnormally dilated blood vessels and retinal bleeding. One month after the first IVR, there was most of resolution of subretinal fluid and regression of abnormal vascular dilation (**c**). The boy underwent 3 IVR, 1 laser photocoagulation, and 1 cryoablation sessions.After 3 months,there was dissolution of abnormal vascular and absorption of subretinal fluid (**d**)

**Fig. 2:**
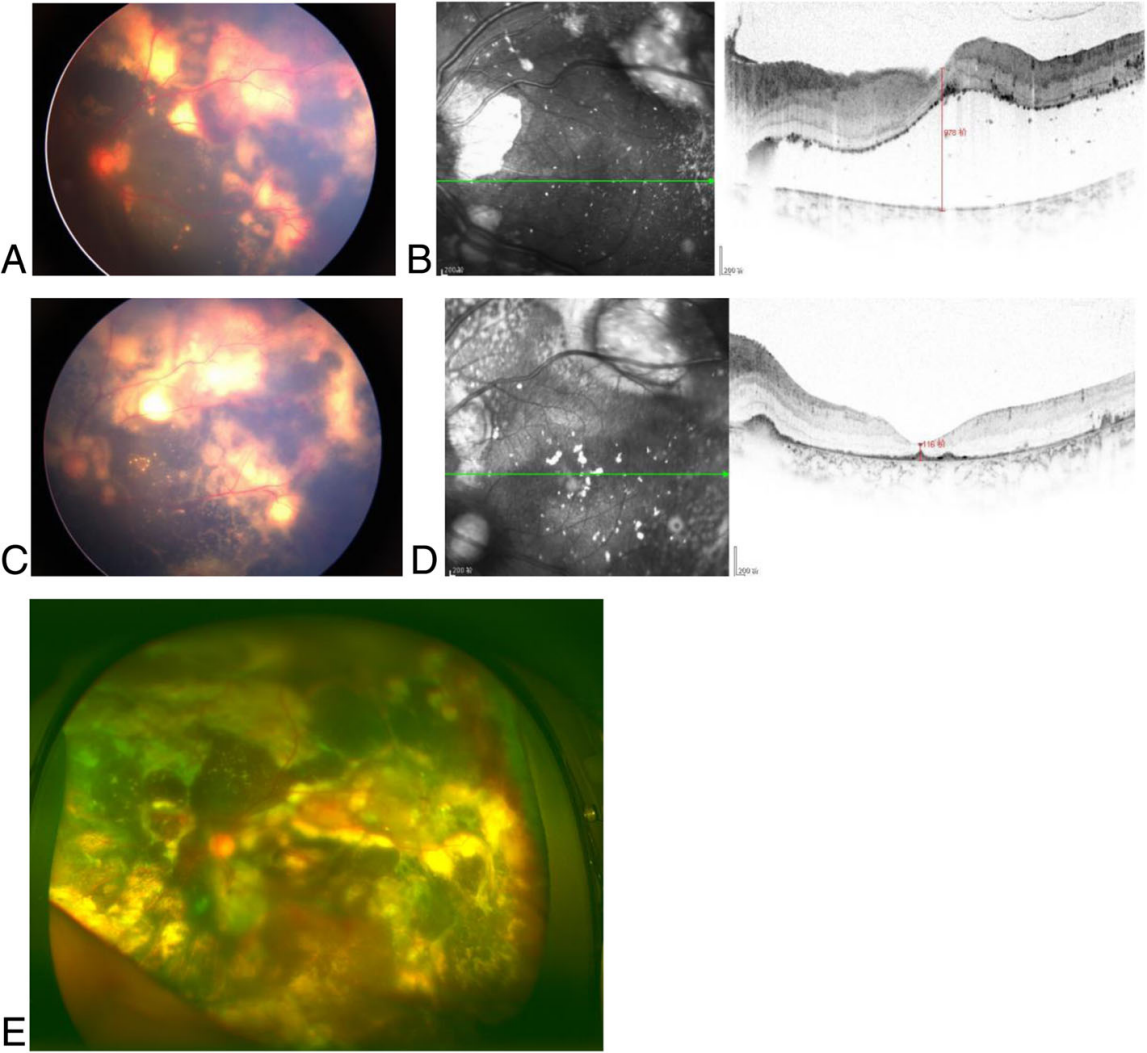
A 9-year-old boy with Coats’ disease of left eye,stage 3A2 .Preoperative of IVR, the Retcam imaging of fudus shows retinal detachment (**a**),macular exudative detachment was 978 μm in OCT (**b**). After 3 IVR,the imaging (**c**, **d**) showed the absorption macular area of the subfluid. Retcam image of 18 months after 3 times intraocular photocoagulation (**e**)

Patients were treated a total of 1 to 5 times for an average of 2.82 ± 0.95 times per patient. 1 patient (5.88%) received IVR one time,5 patients (29.41%) received two times,8 patients (47.06%) received three times, 2 patients (11.76%) received four times and 1 patient (5.88%) received five times. All patients had different degrees of subretinal fluid absorption, and some of the regression of abnormal vascular dilation (Fig 1). OCT and ocular B-ultrasonography showed that the preoperative retinal detachment height was 447 ~ 7900 μm, with an average of 2363.88 μm and a median of 1700 (959.00, 2640.00) μm. After treatment, there was a retinal detachment height of 116 ~ 2380 μm, with was an average of 897.18 μm and median of 779 (361.50, 1157.00) μm. There was a significant difference between the preoperative and postoperative retinal detachment heights according to the 2-related samples Wilcoxon signed rank test (z = 3.517, *p* = 0.000) (Fig 2).

All patients were successfully treated with laser after IVR,12 patients (70.59%) received one laser treatment, and 5 patients received laser treatment more than 2 times.; 3 patients (17.65%) needed retinal cryotherapy as a supplement. No ocular or systemic complications were observed during follow-up.

The intraocular pressure ranged from 8 ~ 19 mmHg with a mean of 11.71 ± 2.95 mmHg. The postoperative IOP was 8 ~ 17 mmHg with a mean of 11.06 ± 0.54 mmHg. There was no statistically significant difference (*t* = 1.580, *p* = 0.135) between the preoperative and postoperative intraocular pressures.

## Discussion

With the occurrence of retinal detachment in patients with Coats’ disease, retinal hypoxia can occur, resulting in increased VEGF in the subretinal fluid and vitreous cavity. Vascular VEGF has been shown to cause telangiectasia, microvascular occlusion, microaneurysms, and, consequently, vascular leakage, which promotes exudation [4, 5, 8]. Some studies have demonstrated that elevated concentrations of VEGF can be detected in the subretinal fluid, vitreous cavity, and aqueous humor in Coats’ disease [4, 8, 9]. Moreover, intravitreal injection of anti-VEGF drugs has been reported to promote subretinal fluid absorption and reattachment of the retina in Coats’ disease. Therefore, this approach represents a new option for treating serious case of Coats’ disease higher than stage 3.

At present, there are few studies on the treatment of Coats’ disease using anti-VEGF drugs. The treatment design of this study mainly followed the principles of minimizing the trauma to patients and achieving the treatment goals with minimal treatment interventions.

This study included 17 patients with Coats’ disease classified higher than stage 3; these patients had severe retinal detachment that could not be effectively treated with retinal laser photocoagulation and cryotherapy alone. Therefore, we first treated the patients with IVR. After retinal reattachment or most of the subretinal fluid was absorbed, patients underwent retinal photocoagulation or retinal cryotherapy. Compared with traditional therapies, including scleral buckling, subretinal fluid drainage, vitrectomy, and gas or silicone oil filling surgery, this approach is simple, safe, effective and involves fewer complications.

Our study showed that the average IVR treatment number was 2.68 in the participants, Only 3 patients needed retinal cryotherapy as a supplement. Retinal cryotherapy effectively damages abnormal retinal vessels. However, some studies have shown that cryotherapy may increase epiretinal membrane formation, retinal traction and even subretinal exudation. Therefore, retinal laser photocoagulation should be performed rather than cryotherapy.

In this study, we chose the retinal detachment height of the macular as an important parameter of drug efficacy because the macular center is easy to locate and observe by OCT. The retinal detachment of all patients was significantly lower after IVR (*p* = 0.000). Although most Coats’ disease cases show peripheral retinal vascular lesions, we observed cases with peripheral retinal shallow detachment in this study after IVR and laser treatment. However, retinal cryotherapy can easily be performed in these cases because of the reduction in retinal detachment.

The results of this study showed that 7 (41.18%) patients had improved visual acuity after IVR treatment. Because ranibizumab treatment could help the exudation under the macular area to absorb rapidly and decreased the macular thickness, and the patient’s visual function can be effectively preserved using this approach. But not all cases are so, the visual acuity of patients without exudation of cholesterol crystals under the macular area could be improved after IVR, while the visual acuity in patients with cholesterol crystals deposited in the macular area was not significantly improved.

Although VEGF expression was detected in the vitreous and subretinal fluid in patients with severe retinal detachment, there have been few reports of neovascularization, and evidence of neovascularization was not observed with retinal angiography.

**Fig. 3:**
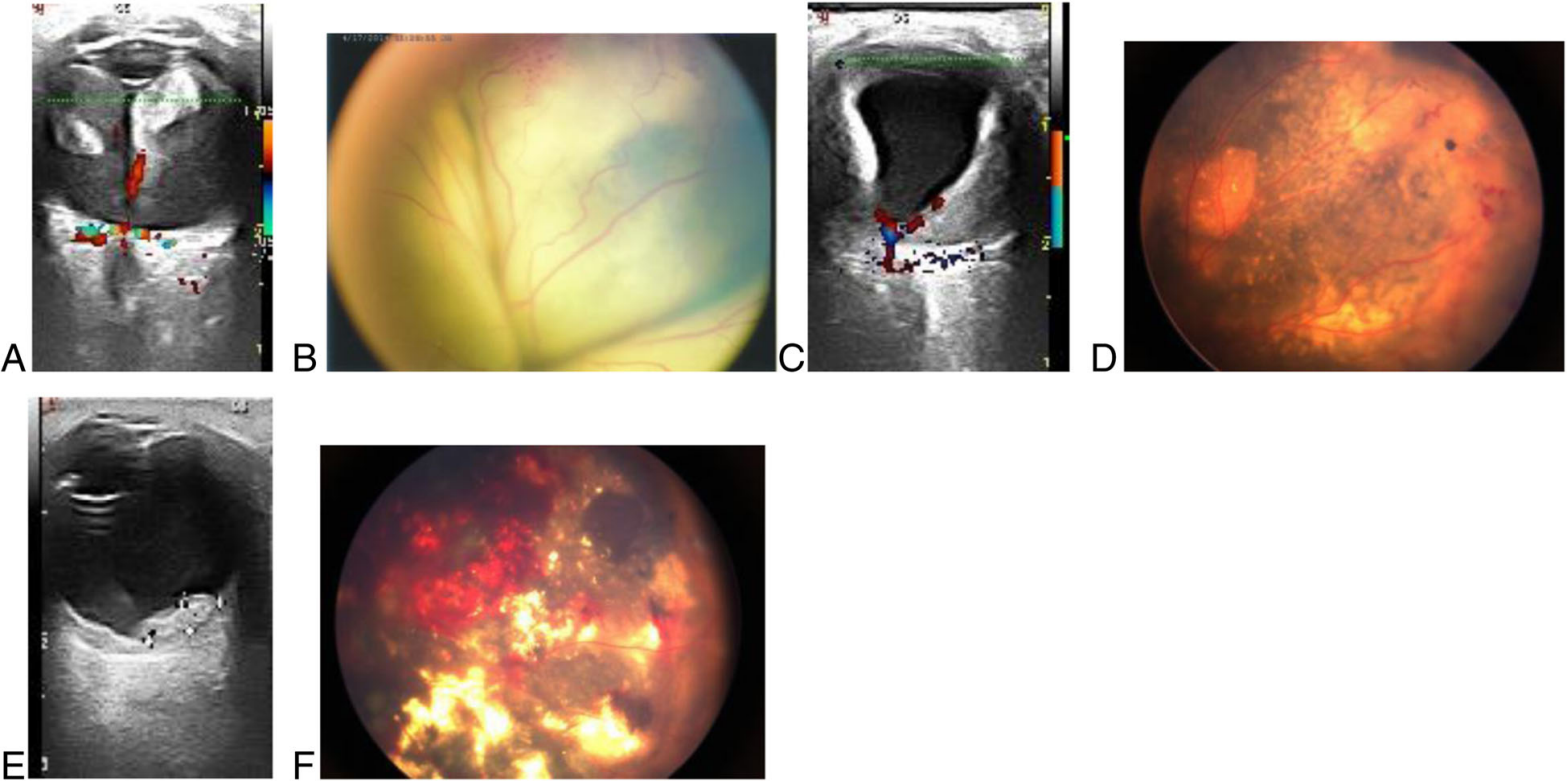
3-year-old boy with Coats’ disease underwent 3 IVR and 1 laser sessions.The preoperative Retcam images and Color Doppler ultrasound images showed total retinal detachment at baseline (**a**, **b**). After 1 month and 3 months of treatment,the Retcam images and Color Doppler ultrasound images showed absorption of Subretinal effusion gradually (**c**, **d**), and reattachment of retinal (**e**, **f**)

In our study, two patients with total retinal detachment showed complete improvement after IVR alone(Fig 3). Moreover, we found that the more severe the retinal detachment, the better the initial response to drugs; therefore, we speculate that for exudative retinal detachment caused by Coats’ disease, the VEGF-induced increase in vascular leakage may be an important cause of retinal detachment. Additionally, this may serve as an effective indication for anti-VEGF drug treatment. However, the retinal detachment of these patients was greater. Some patients also showed spherical retinal detachment that almost reached the lens; therefore, intravitreal injection should be performed carefully to avoid creating iatrogenic retinal holes.

Although anti-VEGF therapy does not cure Coats’ disease, the absorption of subretinal fluid after treatment allows for subsequent laser or cryoablation treatment. Laser treatment for Coats’ disease is an effective approach because the laser can reduce the formation of abnormal blood vessels and the high permeability of the aneurysmal dilatation. In this study, 12 patients received one laser treatment after the anti-VEGF injection, 4 patients received more than 2 laser treatments, and only 3 patients required retinal cryotherapy as a supplement. Although cryotherapy is an effective treatment for abnormal retinal vascular damage, cryotherapy will increase the proliferation of the retina and the occurrence of traction. High levels of cryotherapy will unfortunately increase the leakage of subretinal fluid. Therefore, cryotherapy should be minimized. In this study, the proportion of patients who required retinal cryotherapy was small, which was due to the absorption of subretinal fluid after anti-VEGF therapy. For areas with a poor laser response, cryotherapy can be adequately supplemented, which greatly reduces the need and dose of retinal cryoablation. Eventually, all patients showed preservation of eye shape during follow-up.

In our study, there were no serious complications during the follow-up period, such as endophthalmitis, retinal holes, vitreous hemorrhage or cataracts. A few reports have shown evidence of vitreoretinal fibrosis and retinal detachment after intravitreal injection of anti-VEGF agents, but these were not observed in the present study [10, 11].

This study had several limitations. First, our study group was small, which may have resulted in selection bias. For example, we did not observe vitreous proliferation or traction. Thus, subsequent studies should include a larger sample size. Second, although the average follow-up time in our group was 24.12 months, this duration is not sufficient to observe the chronic course of Coats’ disease. Therefore, our study had limitations in assessing the long-term effects and side effects of these treatments.

## Conclusion

In conclusion, Our study showed that anti-VEGF treatment combination of laser coagulation and cryotheraphy on patients with type 3 Coat’s disease was safe and effective, and it may improve the prognosis of patients with visual acuity deficits. To support anti-VEGF treatment as an adjunctive therapy, a larger sample size with longer follow-up period should be included in a multicenter study to standardize the treatment approach.

## Abbreviations

CDI: Color Doppler imaging
FFA: Fluorescein fundus angiography
IOP: Intraocular pressure
IVR: Intravitreal ranibizumab
OCT: Optical coherence tomography
VEGF: Vascular endothelial growth factor
